# The Prevalence of Obesity Among Employees of a Tertiary Healthcare Organization in Saudi Arabia and Its Impact on the Organization

**DOI:** 10.7759/cureus.26834

**Published:** 2022-07-14

**Authors:** Kossay Elabd, Loay Basudan, Khaled Alabduljabbar

**Affiliations:** 1 Family Medicine, King Faisal Specialist Hospital and Research Centre, Riyadh, SAU

**Keywords:** productivity, impact, hospital, healthcare worker, obesity

## Abstract

Background: Obesity is a significant risk factor for multiple diseases such as diabetes mellitus and cardiovascular diseases. Many healthcare organizations worldwide have identified a high prevalence of obesity among their employees. In this study, we are looking at the prevalence of obesity among the employees of our healthcare organization and its impact on the employees' productivity and if its current prevalence is costly to the employer.

Methods: This is a non-interventional cross-sectional study conducted at King Faisal Specialist Hospital and Research Centre in Riyadh, Saudi Arabia. Data on the current employees were obtained from medical records. We investigated a random sample of employees who worked in the hospital for at least one year between January 1, 2021 and January 1, 2022. We explored the prevalence of obesity among hospital employees at the beginning of their employment and its current prevalence. In addition, we aimed to investigate the effect of having employees suffering from obesity on their productivity and their utilization of healthcare during their employment period.

Results: We identified that our hospital has relatively younger healthcare workers and more employees who are suffering from obesity than in different other countries. The percentage of obese male and female employees was comparable. We had a relatively high increase in the prevalence of obesity among our employees during the last few years. We found a higher number of obesity-related medical problems, more office visits, more sick leaves, and more medications prescribed for overweight and employees who are suffering from obesity compared to those with normal weight.

Conclusion: Healthcare workers suffering from obesity had lower productivity and they had higher utilization of healthcare. Therefore, employers should investigate the prevalence of obesity in their organization and implement diverse strategies to prevent and manage this issue to help their employees have better health and, at the same time, be more productive and lower their utilization of healthcare.

## Introduction

Obesity is a well-known risk factor for many chronic diseases, including type 2 diabetes mellitus, hypertension, dyslipidemia, and knee and spinal osteoarthritis, in addition to atherosclerotic cardiovascular diseases such as coronary artery disease and cerebrovascular diseases [1]. A high prevalence of obesity has been identified among healthcare workers in different countries such as the United Kingdom [2]. The body mass index (BMI) is a measure that uses the person's height and weight to work out what a healthy weight is for that person. Its calculation divides an adult's weight in kilograms by his height in meters squared.

 The World Health Organization (WHO) states that the ideal BMI for an adult ranges from 18.5 to 24.9 kg/m^2^. According to the WHO, BMI which is between 25 and 29.9 kg/m^2^ is in the overweight range, and a BMI ≥ 30 kg/m^2^ means obese. WHO also subdivides obesity into three classes: class 1 - BMI 30 to 34.9 kg/m^2^, class 2 - BMI 35 to 39.9 kg/m^2^ and class 3 (severe obesity) - BMI ≥40 kg/m^2^ [3].

There is a high prevalence of physical inactivity among Saudi Arabian people [4]. Physical inactivity, long working hours and work-related stress are important factors contributing to the high prevalence of obesity and obesity-related morbidity among healthcare workers [5]. With the recent increase in working from home and the increase in fast food delivery services which many employees have been using recently, it will be no surprise that the obesity problem is set to get worse in the next few years.

Many studies in different countries have shown that the high prevalence of obesity among employees was associated with more frequent and more expensive episodes of care than in worksites with low obesity prevalence [6]. Also, many studies have shown a higher rate of sick leaves among obese people [7].

To the best of our knowledge, no previous study has looked at the prevalence of obesity and overweight among healthcare workers in Saudi Arabia. In this study, we tried to estimate the cost healthcare workers with obesity impose on their employers. We did this by checking how many sick leaves they took, medications they were prescribed, and healthcare encounters they needed for obesity-related medical problems during the one year of the study.

 Different methods have been tried to address obesity in the workplace [8]. In this paper, we are discussing different strategies that can be applied to improve the well-being of healthcare workers to help them follow a better lifestyle to reduce the risk of obesity and its related complications.

## Materials and methods

This is a non-interventional, cross-sectional quantitative study investigating the prevalence of obesity and obesity-related morbidities among employees working at King Faisal Specialist Hospital and Research Centre in Riyadh for at least one year between January 1, 2021 and January 1,2022. It also examines the impact of obesity and its related problems on the employees' productivity and the costs the hospital has to bear to look after their health. This was done by reviewing the number of sick leaves needed that year, the number of obesity-related medical issues, the number of healthcare visits, and the number of medications prescribed for obesity-related medical problems that year.

Given that the current total number of employees at King Faisal Specialist hospital is 12,500, the sample size was calculated and was found to be 320. We collected a random sample of 500 hospital employees. We included employees from every department in the hospital who are 18 years and above and who have worked at the hospital for at least one year, between January 1, 2021 and January 1, 2022. We excluded those with other health issues, such as cancer, that can increase the employee's need for medical care and those with incomplete data. Therefore, we included 322 employees in our study. The data were collected from these employees' electronic medical records.

Data were statistically analyzed using the SPSS software version 26.0 by IBM. Descriptive statistics for continuous variables were reported as mean with standard deviation (SD) and categorical variables as frequencies and percentages. We also tested the null hypothesis for continuous variables using t-test and ANOVA, while we used the Chi-square test to compare categorical variables. Parametric person correlation was used to test the correlation between different variables. We set the level of statistical significance at p < 0.05.

The research project was conducted in accordance with the ethical principles set out in the Declaration of Helsinki (2000), WHO Guidelines for Ethical Committees for the Review of Biomedical Research (2000), and International Ethical Guidelines for biomedical research involving human subjects (2002) and the policies of the Research Advisory Committee (RAC) at King Faisal Specialist Hospital and Research Centre, as well as the laws of the Kingdom of Saudi Arabia. The research ethics committee (REC) at King Faisal Specialist Hospital and Research Centre has reviewed and approved the research and was given RAC number 2221012 on January 27, 2022.

## Results

**Figure 1:**
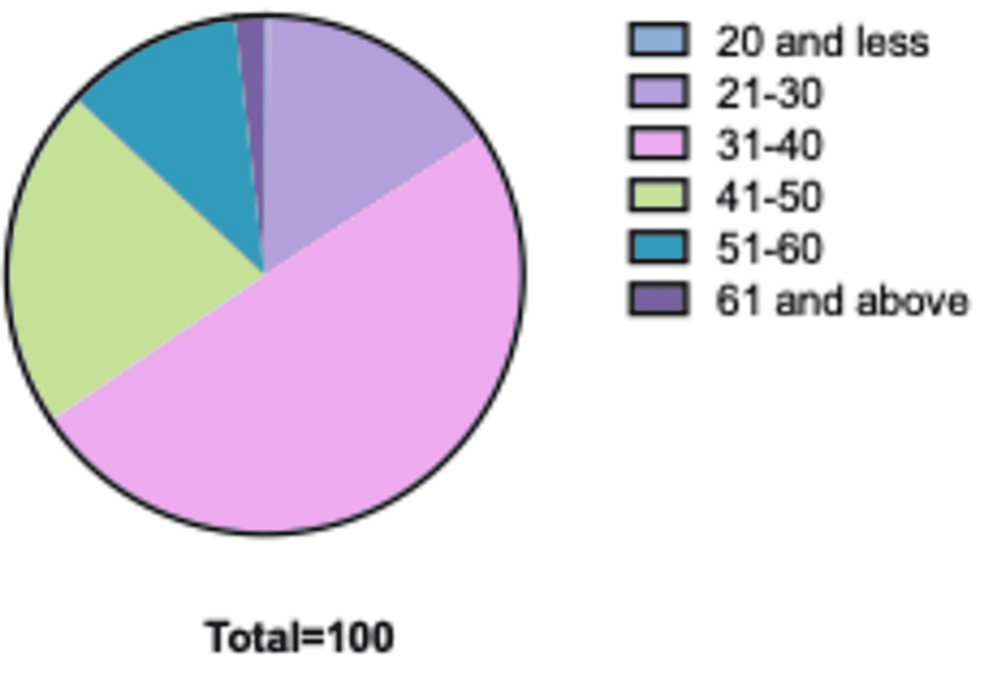
Percentage of age group.

We analyzed the data of 322 employees. We found that 174 were males (54%) and 148 were females (46%). Most of the employees in our sample were between 31 and 40 years old (49%). The percentages of age distribution among our sample are presented in Table 1 and Figure 1.

**Table 1:** Age distribution among the study sample.

Age	Frequency	Percent
20 and less	1	0.3
21 - 30	50	15.5
31 - 40	160	49.7
41 - 50	69	21.4
51 - 60	36	11.2
61 and above	6	1.9
Total	322	100.0

The average duration of employment was 10.19 years (S.D. 6.433) for employees with normal weight and 9.58 years (S.D. 5.273) for employees with obesity. We looked at the prevalence of obesity among our sample of employees during 2021 and we also calculated the percentage of employees in our sample who were obese at the start of their job in the hospital. At the start of employment, 23% of the female and 20.2% of the male employees were obese. In 2021, 36.8% of the female employees were obese, and 38.7% of the males were obese. There was no statistical difference between genders in the prevalence of obesity at the start of employment (p-value 0.551) or in 2021 (p-value 0.725). At the beginning of work, 25% of the Saudi and 16.8 % of non-Saudis employees were obese, but we found no significant statistical difference (p-value of 0.076). In 2021, 32.4% of the non-Saudi employees were obese, and 42% of the Saudi employees were obese but also had no statistical difference (p-value of 0.08). Among those who were obese during 2021, 26.2% had obesity class-1, 8.5% had obesity class-2, and 3.2% had obesity class-3. 21.1% of the employees had documented evidence that they perform some form of a weekly exercise, and 18.4% were currently smoking.

The number of sick leaves taken during the one-year study period by employees with normal weight was 1.68 (S.D. 2.49) per employee, while employees with obesity took 2.05 (S.D. 2.655) per employee that year. We found some degree of correlation between obesity and the number of sick leaves (correlation coefficient 0.138, p-value 0.014)

**Figure 2:**
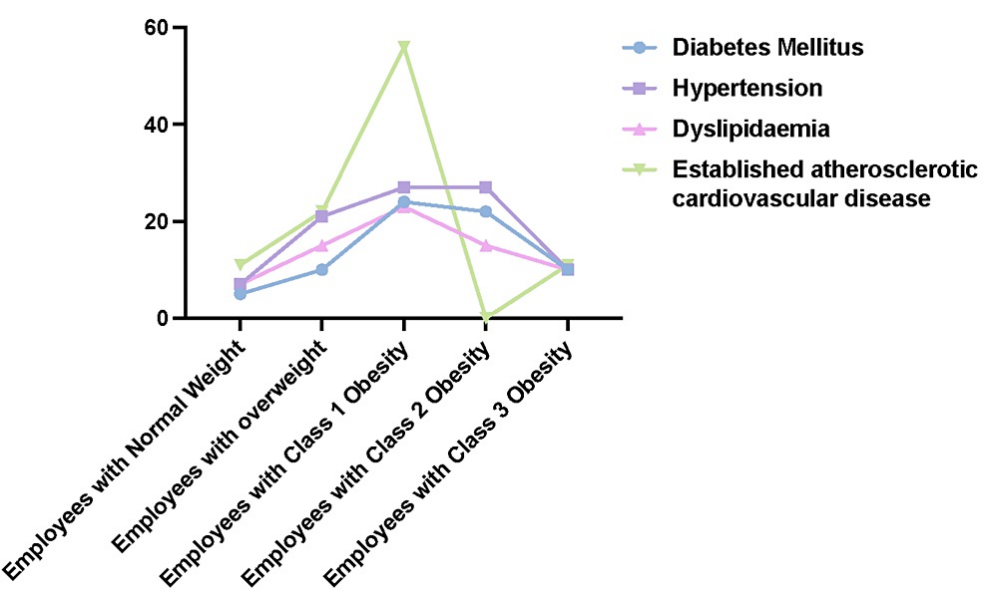
The percentages of different obesity-related medical problems among employees of normal weight and those with varying degrees of obesity.

**Figure 3:**
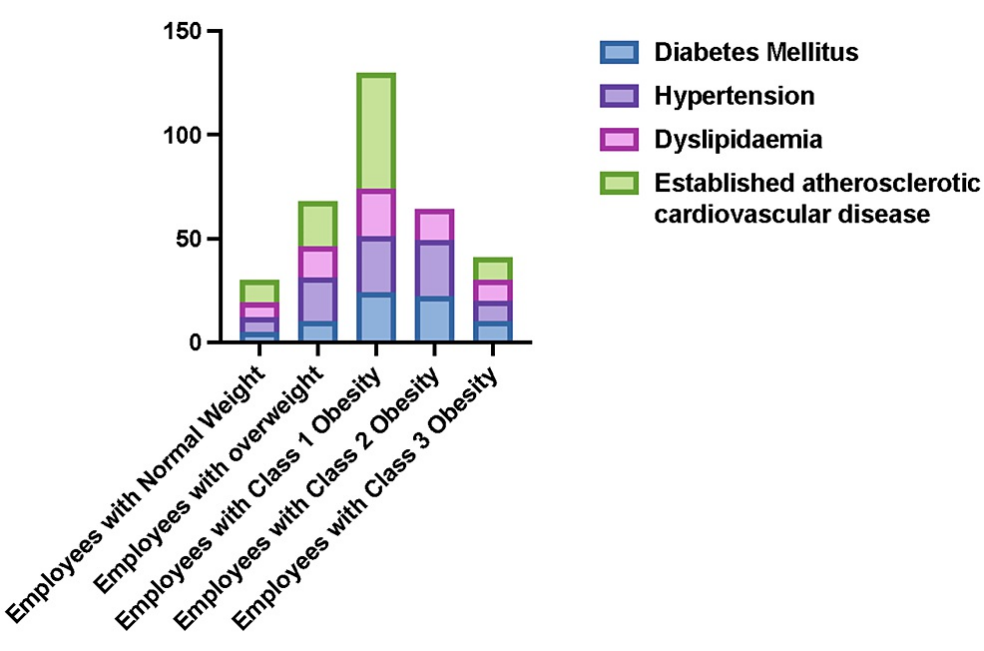
The frequencies of different obesity-related medical problems among employees of normal weight and those with varying degrees of obesity.

In our sample, some of our employees were diagnosed with different medical problems related to obesity, including type 2 diabetes mellitus, hypertension, dyslipidemia, and atherosclerotic cardiovascular diseases. Table 2 and Figures 2, 3 show the percentages of these medical problems among employees of normal weight and those with different degrees of obesity.

**Table 2:** The percentages of different obesity-related medical problems among employees of normal weight and those with varying degrees of obesity.

	Diabetes Mellitus	Hypertension	Dyslipidaemia	Established atherosclerotic cardiovascular disease
Employees with normal weight	4.7%	7.1%	7.1%	11.1%
Employees with overweight	10%	20.9%	14.5%	22.2%
Employees with class 1 obesity	24.1%	26.5%	22.9%	55.6%
Employees with class 2 obesity	22.2%	26.9%	14.8%	0%
Employees with class 3 obesity	10%	10%	10%	11.1%

The number of medications prescribed in one year for hypertension, diabetes, dyslipidaemia or knee or spine osteoarthritis was 0.59 (S.D. 1.164) per employee for normal-weight employees and 1.13 (S.D. 1.851) for employees with obesity. We also found some correlation between the number of prescribed medications and obesity (correlation coefficient 0.208, p-value 0.000).

The number of obesity-related healthcare service encounters, including hospital admissions or outpatient clinic appointments, in 2021 for normal-weight employees was 1.15 (S.D. 1.636) per employee and for employees with. obesity was 1.47 (S.D. 1.805) per employee. We also found some correlation between these encounters and obesity (correlation coefficient 0.187, p-value 0.001).

## Discussion

Forty-nine percent of the employees are between 31 and 40 years old. This makes our employees relatively younger compared to other countries. For example, in the UK, 42.3% of the National Health Service (NHS) staff are between 46 and 65 years old [9]. This can be explained by the relatively younger retirement age in Saudi Arabia, set at 60 for men and 55 for women. Moreover, workers in Saudi Arabia could retire at any age if they had contributed for at least 300 months (25 years) [10]. In the UK, the retirement age is 65 years old [11]. In the USA, the retirement age is 66 years and two months for people born in 1955, and it gradually rises to 67 for those born in 1960 or later [12]. This could potentially mean that our hospital is losing older employees who have more experience through earlier retirement, and these retiring staff is also losing due to not staying active at work, which can negatively affect their health and social wellbeing.

The current prevalence of obesity among our hospital employees is relatively higher than what has been seen in some European countries like the UK. In our study, we found that during the year 2021, 36.8% of the female employees were obese, and 38.7% of the males were obese. In the UK, a study found that about 25% of nurses and 14% of other healthcare professionals were obese [2]. On the other hand, studies from the middle east for example in the United Arab Emirates a study has found an obesity prevalence of 47.3%, which is higher than we found in our hospital [13].

Over the average employment period of our sample (10.19 years, S.D. 6.433), we had a 13.8% in obesity prevalence among females, 18.7% among males, 17% among Saudi employees and 15.6% among non-Saudi employees. The percentage of employees currently smoking in our hospital was almost like in other countries like the UK [14]. One-fifth of the employees in our sample documented some level of physical activity. In most cases, this activity was some walking during the working hours they had to do as part of their duties.

To investigate the influence of this increasing obesity prevalence on our healthcare organization, we compared the number of diagnosed obesity-related medical problems between employees with obesity and those with a normal weight, the number of prescribed medications taken for obesity-related medical problems, and the number of sick leaves taken per employee between January 1, 2021 and January 1, 2022.

There was a higher sick leave rate among overweight or obese employees. Although this difference was not statistically significant (p-value of 0.054), we found some correlation between obesity and the number of sick leaves (correlation coefficient 0.138, p-value 0.014).

Furthermore, we found a higher prevalence of obesity-related medical problems such as type 2 diabetes, hypertension, dyslipidemia, and cardiovascular atherosclerotic diseases, especially among those with class 1 obesity and, to a lesser extent, those who are overweight and those with class 2 obesity. We also found some degree of correlation, although not very high, between obesity and the number of obesity-related healthcare service encounters, including hospital admissions or outpatient clinic appointments, during the one year of the study and with the number of medications prescribed in that year for hypertension, diabetes, dyslipidemia or knee or spine osteoarthritis. Employees with obesity were prescribed twice as much medication as employees with normal weight. This potentially means that employees with obesity cost the healthcare organization more than those with normal weight.

Obesity in the workplace has always been an issue affecting employees and their employers [2]. Addressing this issue is very important and can serve the employer's economic interests. It is essential for healthcare organizations, like our hospital, to play a central role in helping their employees have a better lifestyle and maintain healthy body weight. This can start by assessing the impact of obesity on their employees' health and performance at work, then using best practices to build healthy workplace environments and communicating with employees effectively and sensitively on the topic of obesity.

Different methods have been implemented by employers to help their employees achieve and maintain a healthy weight and lifestyle. For example, starting weight management initiatives in the workplace with the help of physicians, nurses, and dietitians to encourage employees to change their lifestyle with a healthy diet, regular exercise and stop unhealthy habits such as smoking. Providing healthy food options in the hospital canteens and on-site vending machines. Encouraging employees to have regular physical activity and exercise through, for example, motivating staff to use stairs instead of lifts, giving employees frequent breaks to avoid long periods of sitting time and encouraging them to utilize these breaks for some physical activity, sponsoring employees who are engaging in fitness and health challenges, providing the employees with a discounted or free gym membership. Some employers have promoted standing desks to encourage physical activity and decrease prolonged sitting.

According to the best of our knowledge, our study is the first study in Saudi Arabia that looked in such detail at the problem of obesity in the workplace and its associated medical problems and the impact of it on the employer in terms of the cost of healthcare needed to look after the obese employees and the number of sick leaves needed by these employees.

In our study, we did not separately investigate the prevalence of obesity-related medical problems in older employees, who might have a higher rate than what we found in the relatively younger employees. We also only looked at the number of clinical encounters, sick leave and prescribed medication needed in one year, which might not reflect the actual number that might have happened over the total period of employment. Moreover, some of our employees, including the obese ones, seek medical help from other health facilities, which means that we do not have complete information about their medical problems. Additionally, not all of our sample employees had precise information about their smoking status or how much physical activity they engage in.

Future studies may consider investigating the prevalence of obesity and its related problems in different age groups. They can explore the cost of employing obese employees and compare it with the expenses employers might need to bear to provide their employees with strategies to help them prevent or control obesity.

## Conclusions

Our hospital’s employees are relatively younger, and we have a higher prevalence of obesity than other countries like the UK. We found that employees who suffer from obesity are requesting more sick leaves, more frequently utilizing healthcare services, and being prescribed more medications for obesity-related medical problems. In addition to affecting the employees' health, obesity impacts the employees’ efficiency and costs the employers more. Therefore, it is essential for employers to recognize the effect of obesity on their employees and different strategies should be implemented in the workplace to prevent and treat obesity by encouraging their employees to lead a healthy lifestyle.
